# Structure-based inhibitors targeting the alpha-helical domain of the *Spiroplasma melliferum* histone-like HU protein

**DOI:** 10.1038/s41598-020-72113-4

**Published:** 2020-09-15

**Authors:** Yuliya K. Agapova, Dmitry A. Altukhov, Vladimir I. Timofeev, Victor S. Stroylov, Vitaly S. Mityanov, Dmitry A. Korzhenevskiy, Anna V. Vlaskina, Eugenia V. Smirnova, Eduard V. Bocharov, Tatiana V. Rakitina

**Affiliations:** 1 National Research Center “Kurchatov Institute”, Akad. Kurchatova pl., 1, Moscow, 123182 Russian Federation; 2 Federal Scientific Research Center “Crystallography and Photonics”, Russian Academy of Sciences, Leninskii Pr., 59, Moscow, 119333 Russian Federation; 3 N. D. Zelinsky Institute of Organic Chemistry, Russian Academy of Sciences, Leninskii pr., 47, Moscow, 119991 Russian Federation; 4 D. Mendeleyev, University of Chemical Technology of Russia, Miusskaya sq., 9, Moscow, 125047 Russian Federation; 5 Shemyakin–Ovchinnikov Institute of Bioorganic Chemistry, Russian Academy of Sciences, ul. Miklukho-Maklaya, 16/10, Moscow, 117997 Russian Federation; 6 Moscow Institute of Physics and Technology (State University), Institutskiy Per., 9, Dolgoprudny, Moscow Region, 141701 Russian Federation

**Keywords:** Biochemistry, Biophysics, Computational biology and bioinformatics, Drug discovery, Molecular biology, Structural biology

## Abstract

Here we report bisphenol derivatives of fluorene (BDFs) as a new type of chemical probes targeting a histone-like HU protein, a global regulator of bacterial nucleoids, via its dimerization interface perturbation. BDFs were identified by virtual screening and molecular docking that targeted the core of DNA-binding β-saddle-like domain of the HU protein from *Spiroplasma melliferum.* However, NMR spectroscopy, complemented with molecular dynamics and site-directed mutagenesis, indicated that the actual site of the inhibitors’ intervention consists of residues from the α-helical domain of one monomer and the side portion of the DNA-binding domain of another monomer. BDFs inhibited DNA-binding properties of HU proteins from mycoplasmas *S. melliferum, Mycoplasma gallicepticum* and *Escherichia coli* with half-maximum inhibitory concentrations in the range between 5 and 10 µM. In addition, BDFs demonstrated antimicrobial activity against mycoplasma species, but not against *E. coli*, which is consistent with the compensatory role of other nucleoid-associated proteins in the higher bacteria. Further evaluation of antimicrobial effects of BDFs against various bacteria and viruses will reveal their pharmacological potential, and the allosteric inhibition mode reported here, which avoids direct competition for the binding site with DNA, should be considered in the development of small molecule inhibitors of nucleoid-associated proteins as well as other types of DNA-binding multimeric proteins.

## Introduction

In bacteria, proper assembly of the higher-order nucleoid structure requires the accessory of nucleoid associated proteins (NAPs) including leucine-responsive regulatory protein (LRP), factor for inversion stimulation (FIS), histone-like nucleoid structuring (H-NS), integration host factor (IHF) and histone-like HU protein (HU)^1^. The most abundant protein among these global regulators of DNA topology^2^ was named HU after “histone” and *Escherichia coli* strain U93 used in 1970s for bacterial nucleoid isolation^3^. HU is omnipresent in bacteria and was also found in plastid-bearing *Eukaryota, Euryarchaeota, Thaumarchaeota* and some viruses^4^. It is a small basic dimeric protein with a highly conserved spatial structure^5^, which consists of a compact α-helical domain and a DNA-binding domain containing a saddle-shaped β-sheet and protruding β-ribbon DNA-spanning arms, highly mobile in the absence of DNA^6–9^. In contrast to the majority of *E. coli* DNA-binding proteins recognizing specific DNA sequences (e.g. Lac repressor^10^) and to its closest homolog IHF^11^, HU proteins bind DNA in a non-sequence-specific manner with an enhanced affinity to distorted DNA structures such as forks, junctions, nicks and overhangs^12–16^.

As key NAPs, HU proteins are involved in DNA compaction and supercoiling as well as in the formation of higher order protein complexes, which regulate vital DNA functions including replication, recombination, repair, and transcription with consequent effect on the bacterial transcriptome and proteome^17–21^. HU proteins have been shown to participate in the transcriptional regulation of the genes associated with virulence of *Salmonella enterica serovar Typhimurium*^22^, *Francisella tularensis*^23^, and *Porphyromonas gingivalis*^24^. HU proteins play a role in biofilm formation and overall pathogenesis of *Xanthomonas citri*^25^ and *Cytophaga hutchinsonii*^26^. In *E. coli*, HU proteins control expression of about 8% of genes mostly associated with adaptation and stress response^20^. Deletion of both genes coding the HU heterodimer from the *E. coli* genome is not lethal if other NAPs are preserved^27^. In some other bacteria, e.g. *Bacillus subtilis*^28^, *Streptococcus pneumonia*^29^ and *Mycobacterium tuberculosis*^30^, HU proteins are more essential for cell viability. In general, the importance of HU proteins for bacterial survival depends on the presence of other NAPs, which are able to compensate for the absence of HU. Thus, HU proteins are crucial for the bacteria where they are the only NAPs available, such as Mycoplasma genus (class Mollicutes), the smallest known microorganisms characterized by absence of the cell wall and reduced genome size^31,32^. Mollicutes are parasites of higher Eukaryotes, frequently acting as causative agents of common infections and main contaminants of cell cultures and vaccines. Controlling mycoplasma infections is a serious challenge^33,34^. The high importance of HU proteins for Mycoplasma survival makes them an attractive target for the development of antimycoplasmic agents.

Earlier, inhibition of DNA-binding properties of *M. tuberculosis* HU protein (MtbHU) was achieved using trans-stilbene derivatives, which also affected the growth of the corresponding bacteria, suggesting that MtbHU (as well as any other HU) might be considered as a target for pharmacological intervention^35^. Recently, the same stilbene derivatives were shown to disrupt DNA-binding properties of a viral HU/IHF-homolog (pA104R) from African swine fever virus and inhibit the replication of the virus in primary porcine alveolar macrophages^36^. We followed the design strategy proposed in^35^ for the development of chemical inhibitors of HU protein from mycoplasma *Spiroplasma melliferum* (HUSpm). High-resolution spatial structure of HUSpm (PDB ID 5L8Z^9^) was used for virtual screening and docking, while its structure in solution (PDB ID 5OGU^37^) was employed for the comparative analysis of conformational dynamics observed in the HUSpm dimer upon either independent or sequential addition of short double-stranded DNA and an inhibitor. We found out that bisphenol derivatives of fluorene (BDFs) inhibit DNA binding properties of three model HU proteins (HUSpm, HUα-homodimer from *E. coli* (HUEc) and HU from another mycoplasma species *Mycoplasma gallisepticum* (HUMgal)). BDFs also demonstrated antimicrobial activity against mycoplasma, but not against *E. coli*, which is consistent with a compensatory role of other NAPs in the higher bacteria. Surprisingly, high-resolution heteronuclear NMR spectroscopy complemented with molecular dynamics and site-directed mutagenesis revealed that BDFs do not target the site that had been described for the trans-stilbene derivatives in^35^. Instead of the DNA-binding cleft, BDFs target the side portion of HUSpm that overlaps the dimer interface between the α-helical domain of one monomer and the β-saddle domain of another monomer causing a perturbation of the dimer interface which interferes allosterically with the natural conformational dynamics of the DNA-binding domain and the corresponding efficiency of DNA binding. We believe that this inhibition mode without direct competition for the binding site with natural HU proteins’ ligand (DNA) is of high scientific and pharmacological interest. It is likely that other new HU protein inhibitors with similar properties will be discovered soon, since targeting the HU protein α-helical body may be less challenging than targeting a large and adaptive DNA-binding area.

## Results

### Identification of HUSpm–DNA interaction inhibitors among bisphenol derivatives of fluorene

**Figure 1:**
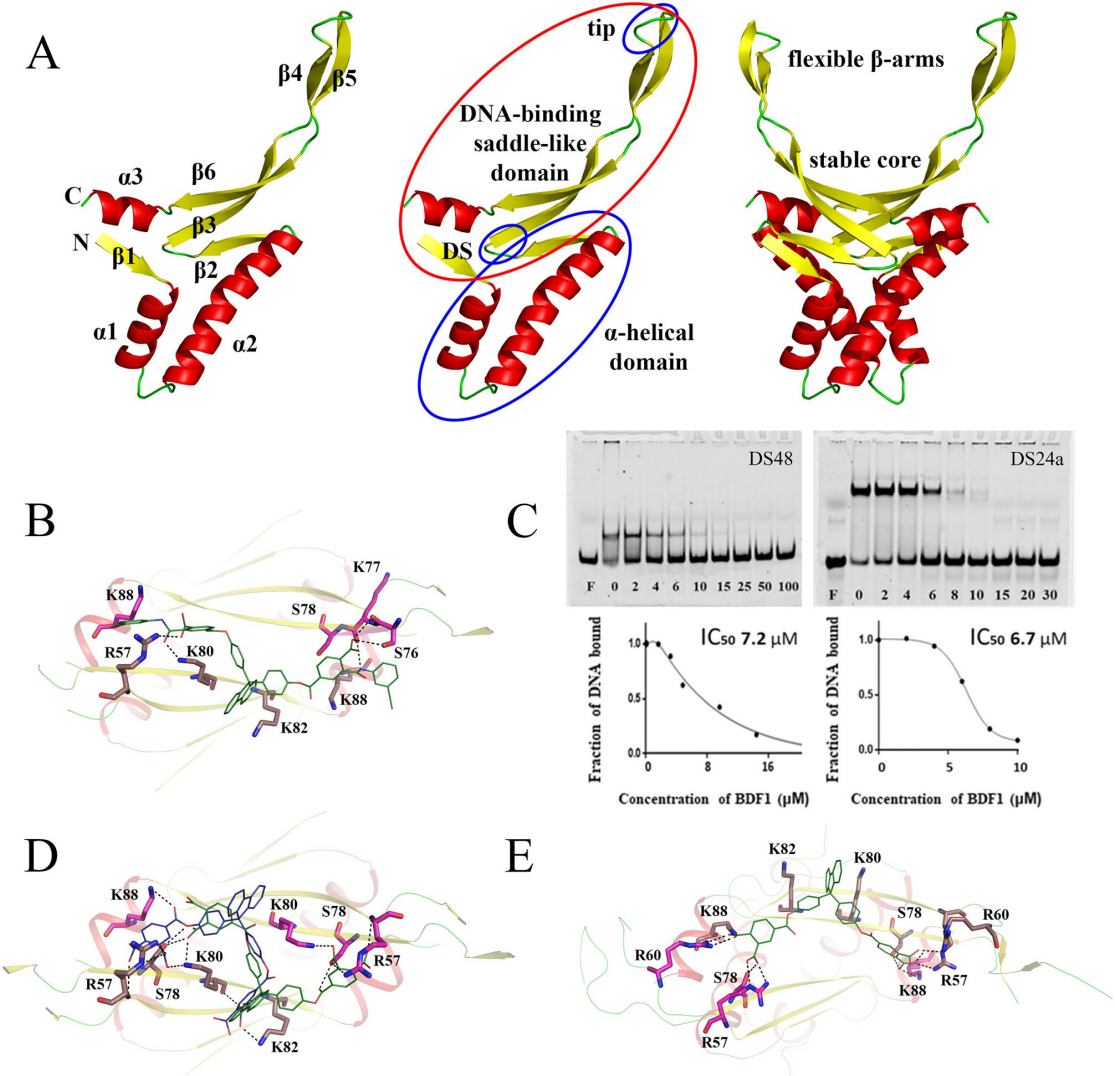
Description of HUSpm domain structure and discovery of HUSpm inhibitors. (**A**) Left to right: secondary structure elements, domains and their subdivisions are shown on cartoon models of HUSpm monomer and dimer. DS—dimerization signal (GFGKF), tip—DNA-intercalating loop (GRNP). (**B**) Model of BDF1 (compound 5 from Supplementary Table S1) binding to the core of the HUSpm DNA-binding domain and polar interactions between BDF1 and positively charged or polar residues of HUSpm. Residues from different monomers are in magenta and beige colour, respectively. (**C**) Inhibition of HUSpm-DNA binding by different concentrations of BDF1, corresponding inhibition curves and IC_50_ values. Two different oligonucleotide duplexes—48 bp DS48 and 24 bp DS24a carrying a single nucleotide insertion in one strand—were used in the assay with similar results. (**D**) Model of BDF4 (Supplementary Table S3) binding to the core of the HUSpm DNA-binding domain in two locations and polar interactions between BDF4 and positively charged or polar residues of HUSpm. Residues from different monomers are in magenta and beige colour, respectively. (**E**) BDF4 position and polar interactions after 1,000 ns MD simulation.

In silico screening by molecular docking and filtering was performed to identify compounds interacting with the core region of the DNA-binding saddle-like domain of HU proteins (Fig. 1A) previously characterized in^35^. High-resolution spatial structure of HUSpm (PDB ID 5L8Z^9^) was chosen as a target for virtual screening of one million compounds from the Vitas-M laboratory library collection (https://vitasmlab.biz/). The screening (see "Methods" for a description) identified a set of five compounds (Supplementary Table S1), further evaluation of which was carried out by an electromobility shift assay (EMSA) performed as described in^5^. In the assay, formation of DNA–protein complexes retards migration of dye-labeled DNA-duplexes during non-denaturing PAGE that gives a shifted fluorescent band. The presence of an inhibitor prevents complex formation and corresponding band shifting.

The first round of EMSA was performed using 100 μM concentration of the compounds and a 48 bp oligonucleotide duplex (DS48) and demonstrated that a bisphenol derivative of fluorene (BDF), referred here as BDF1 (compound 5 in Supplementary Table S1), inhibits HUSpm-DNA binding (Supplementary Figure S1). Positioning of BDF1 in the core region of the HUSpm β-saddle and its polar contacts with amino acids of the DNA-binding domain are shown in Fig. 1B. The BDF1 inhibition curves (Fig. 1C) obtained in EMSA performed with two different oligonucleotide duplexes (DS48 and a 24 bp duplex carrying a single nucleotide insertion in one strand (DS24a)) showed very similar half-maximum inhibitory concentration (IC_50_) values for HUSpm-DNA binding—6.5 ± 0.9 μM and 6.2 ± 0.5 μM, respectively (Supplementary Table S1). The shorter duplex (DS24a) was routinely used in further experiments because of a sharper band of bound DNA at the physiological (150 μM) salt concentration and better separation of bound and unbound DNA during PAGE, which facilitated quantification of EMSA results.

After successful evaluation of BDF1 as a HUSpm-DNA binding inhibitor, we obtained an additional set of six other BDFs found in the screening collection of Chemical Block Ltd (Moscow, Russia, https://www.chemical-block.com) (Table 1) and analyzed their effects on the HUSpm-DNA binding. Two compounds, BDF2 and BDF3, were identified as HUSpm inhibitors by EMSA performed with DS24a, although their IC_50_ values (29 ± 5 μM and 36 ± 6 μM, respectively) were significantly lower than that of BDF1 (Supplementary Table S1 and Figure S2). Comparison of BDF2 and BDF3 with BDF1 suggested that the carboxyl groups of BDF1 play an important role in the binding of the inhibitor to the HUSpm β-saddle core enriched in positively-charged amino acids.

**Table 1:** Inhibition of HU protein DNA-binding by synthesized BDFs.

Compounds	BDF4	BDF5	BDF6
HU protein	IC_50_, μM (average ± SD from three runs of EMSA)		
HUSpm	5.6 ± 0.8	6.3 ± 0.7	8.2 ± 1.1
HUMgal	5.0 ± 0.6	5.9 ± 0.8	8.1 ± 1.0
HUEc	7.5 ± 1.0	7.5 ± 0.9	10 ± 1.3
**Activities of BDF4 against HUSpm mutants, IC**_**50**_**, μM (mean ± SD from three runs of EMSA)**			
HUSpm_R57A	HUSpm_K80A	HUSpm_R88A	HUSpm_K35T
6.3 ± 1.0	5.9 ± 0.9	6.3 ± 1.2	1.8 ± 0.3

Therefore, we decided to obtain three additional BDFs (BDF4, 5 and 6, Supplementary Table S3) using chemical synthesis. The synthesis scheme and the detailed protocols are provided in Supplementary Figure S3 and Supplementary methods, respectively. Given a high degree of similarity, BDF5 was used to replace BDF1, since the latter had been received from the Vitas-M laboratory collection in an insufficient quantity. BDF4 and BDF6 had lower molecular weights (their MWs were 734 and 646 Da vs. MWs 932 and 912 Da for BDF1 and BDF5, respectively) and contained four and two carboxyl groups, respectively. Docking of BDF4 in the core region of the DNA-binding saddle-like domain of HUSpm showed that twofold degenerate center-symmetrical binding poses (Fig. 1D) are possible (ΔG ~ − 9 kcal/mol), and the binding in both positions is similar to that observed for BDF1 (Fig. 1B). However, subsequent MD simulation revealed a more advantageous binding mode (shown in Fig. 1E). In this position, the fluorene was placed between side chains of K82 and K80 of one monomer thus forming pi-cation interactions, while carboxyl groups electrostatically interacted with side chains of the DNA-binding domains’ basic and polar residues R57, R60, S78, and K88 from both monomers.

### Bisphenol derivatives of fluorene inhibit DNA-binding properties of HU proteins from different bacteria as well as mycoplasma cell growth

For further evaluation of the three newly synthesized BDFs (BDF4, 4.4′-[4.4′-(9H-fluorene-9.9-diyl)-bis(4.1-phenylene)]-bis(oxy)-bis(oxomethylene)diphthalic acid; BDF5, 4.5′-[4.4′-(9H-fluorene-9.9-diyl)-bis(4.1-phenylene)]-bis(oxy)-bis(oxomethylene)-bis[2-(m-tolylcarbamoyl)-benzoic acid]; BDF6, 3.3′-[4.4′-(9H-fluorene-9.9-diyl)-bis(4.1-phenylene)]-bis(oxy)-bis(oxomethylene)-dibenzoic acid) we employed two other HU proteins in addition to HUSpm. One protein was from mycoplasma *M. gallisepticum* (HUMgal) and another was HUα-homodimer from *E. coli* (HUEc). As we have reported earlier, the three chosen HU proteins belong to two distant HU clades and have differential specificity patterns towards various DNA structures^5^. Comparison of their amino acid sequences revealed 25% sequence identity for the HUSpm and HUMgal pair, 42% for the HUSpm and HUEc pair, and 23% for the HUMgal and HUEc pair. In addition, HUMgal has a six amino acid N-terminal extension and HUSpm has a two amino acid insertion between α-helices 1 and 2 (Supplementary Figure S4). To assess the effect of the three BDFs on DNA binding properties of the three HU proteins, increasing concentrations of inhibitors were mixed with preset DNA–protein complexes and EMSA was performed and quantified as described in the Methods. DS24a was used in all EMSA experiments.

**Figure 2:**
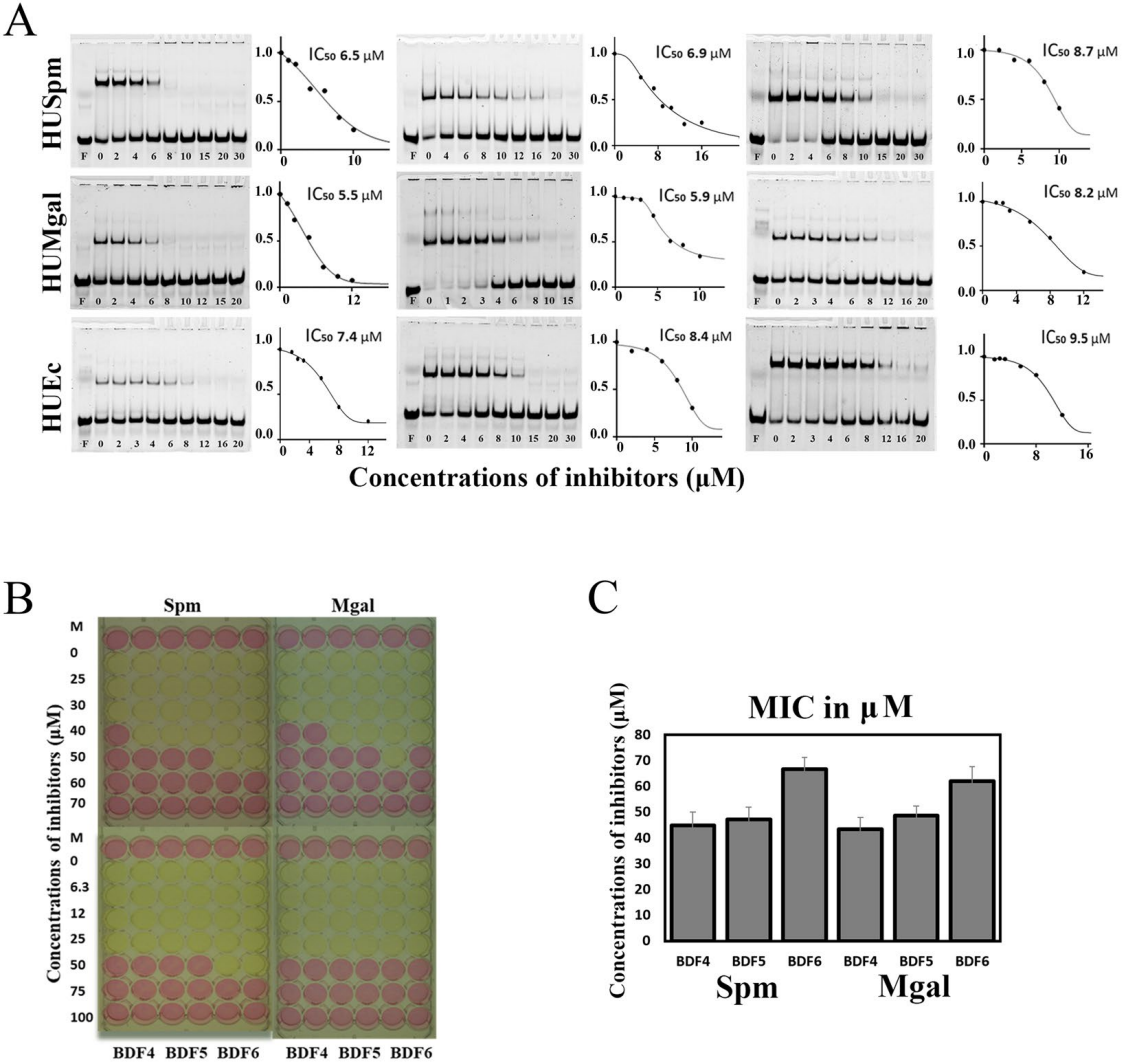
Effect of BDFs on DNA-binding of HU proteins from different bacteria and on mycoplasma cell growth. (**A**) Inhibition of HUSpm, HUMgal and HUEc DNA-binding determined in EMSA performed with DS24a duplex, corresponding inhibition curves and IC_50_ values. (**B**) Effect of BDF4, BDF5 and BDF6 on *M. gallisepticum* and *S. melliferum* growth in culture. The yellow colour indicates the growth of bacteria, pink indicates no growth. MIC was defined as the minimum inhibitory concentration that prevented pink to yellow colour change. (**C**) Graphical representation of the combined results of three independent MIC determinations.

As Fig. 2A shows, all compounds exhibited inhibitory effects against all target proteins with IC_50_ values for DNA binding at micromolar (< 10 μM) concentration ranges (Table 1). In all cases, the observed order of compounds by effectivity of inhibition was consistent: BDF4 > BDF5 > BDF6. The difference between BDF4 and BDF5 (and its prototype BDF1) was not significant, while BDF6 showed slightly higher IC_50_ values.

Since HU proteins are known to be essential for mycoplasma cell viability^31, 32^, we assessed the effects of the inhibitors on *M. gallisepticum* and *S. melliferum* growth in culture. The minimal inhibitory concentrations (MIC) of BDF4, BDF5 and BDF6 for corresponding mycoplasmas were determined in the broth micro-dilution test^38^. The growth of metabolizing organisms causes a change in the pH of the medium leading to a visible color change due to the presence of phenol red in the medium. When the growth of bacteria is inhibited, color change is not observed. The MIC is the lowest inhibitor concentration that prevents the color change at the time when the color change has developed in control (untreated) samples. The MICs were found to be in the range from 40 to 70 μM (Fig. 2B). BDF6 had the worst MIC as compared to either BDF4 or BDF5, whose MICs were not significantly different from each other (Fig. 2C).

No inhibitory effect on *E. coli* culture growth was observed (data not shown) which coincided with the report that *E coli* cells with double knockout of HU heterodimer coding genes were viable and grew well in standard cultivation conditions^20^. In addition, several other factors (e.g. intra-cellular permeability and stability) could contribute to the resistance of highly organized bacteria to HU inhibitors.

### NMR spectroscopy complemented with molecular dynamics suggests that bisphenol derivatives of fluorene target the HUSpm alpha-helical domain

Earlier, we characterized the structural and motional features of HUSpm using nuclear magnetic resonance (NMR) spectroscopy; the backbone and side chain resonance assignments and the secondary structural features derived empirically from backbone chemical shifts were reported in^37^. Since NMR can also be used to study the structure–activity relationships corresponding to specific inhibitors binding to target proteins, here we performed the backbone ^15^N-NMR relaxations experiments to characterize the motional features of HUSpm upon both simultaneous and separate addition of the inhibitor and DNA.

Among the three BDFs described in the previous charter, BDF4 was chosen for NMR study due to its highest activity in all tests and water solubility. Both BDF5 and BDF6 had limited solubility; BDF5 also had higher molecular weight, and BDF6 was less active. To confirm BDF4 binding to the β-saddle domain core of HUSpm, we performed titration of the ^15^N-labelled protein (both in its free form and in complex with the DS14 duplex (Supplementary Table S4)) with different amounts of the inhibitor. ^15^N-HUSpm preparation and conditions of NMR spectroscopy were established in the previous work^37^. The interactions of HUSpm with the short canonical double-stranded DNA (DS14) were also subjected to the NMR study.

**Figure 3:**
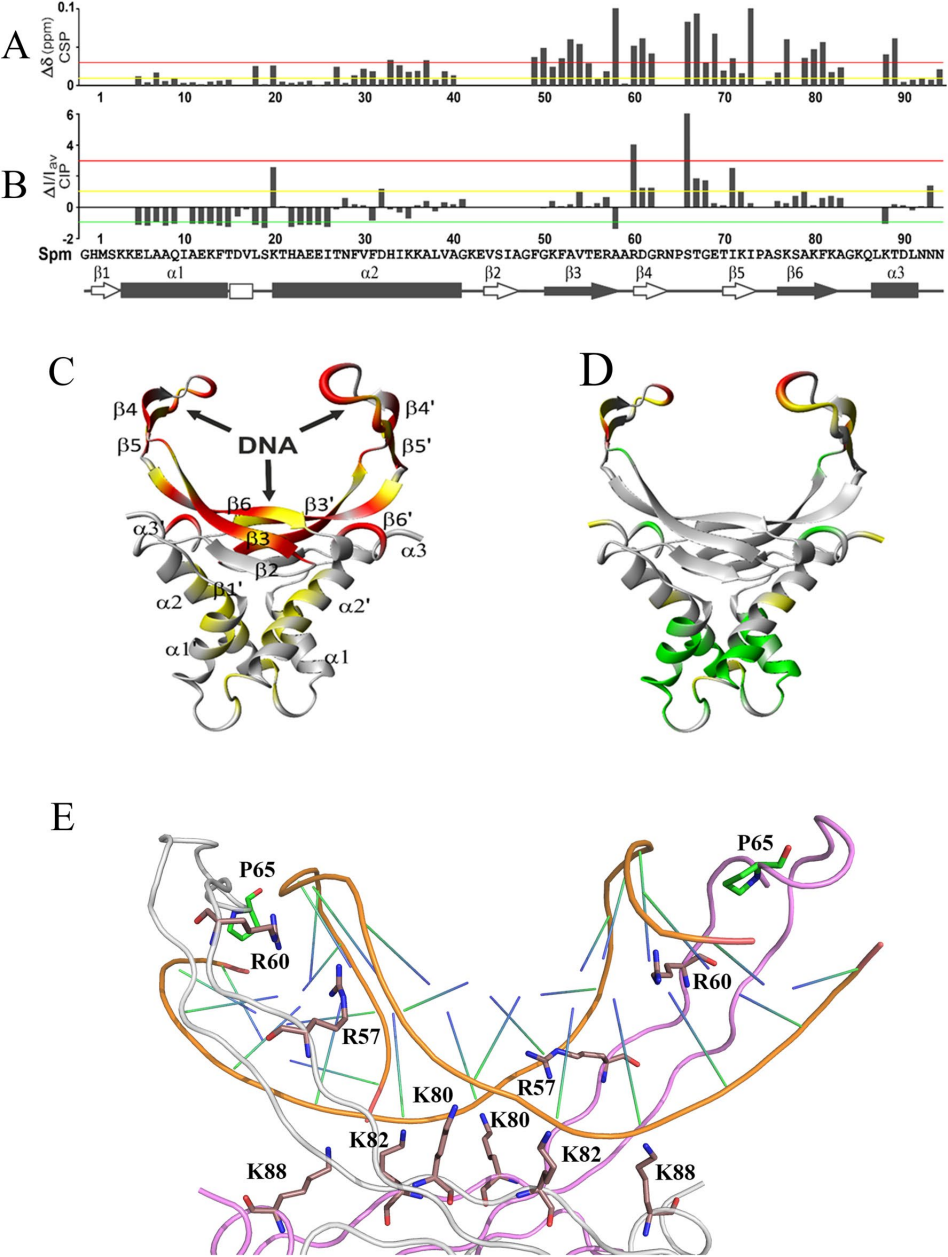
Monitoring of chemical shift perturbations (CSPs) and cross-peak intensity perturbations (CIPs) of backbone amide groups of the HUSpm dimer upon DNA-binding. Weighted CSPs (**A**,**C**) and normalized CIPs (**B**,**D**) of HUSpm residues after binding DS14 shown as diagrams (**A**,**B**) and their color-coded mapping on ribbon models of the HUSpm dimer (**C**,**D**). Green, yellow and red lines in (**A**) and (**B**) are cutoff levels for the color-coding used in (**C**) and (**D**). (**E**) Model of HUSpm interacting with a DS14 duplex after 1,000 ns MD simulation. Two monomers are shown with gray and magenta lines. Basic residues from both monomers interacting with DNA through their side chains are shown in beige sticks; Pro65 intercalating into the DNA minor groove is in green.

**Figure 4:**
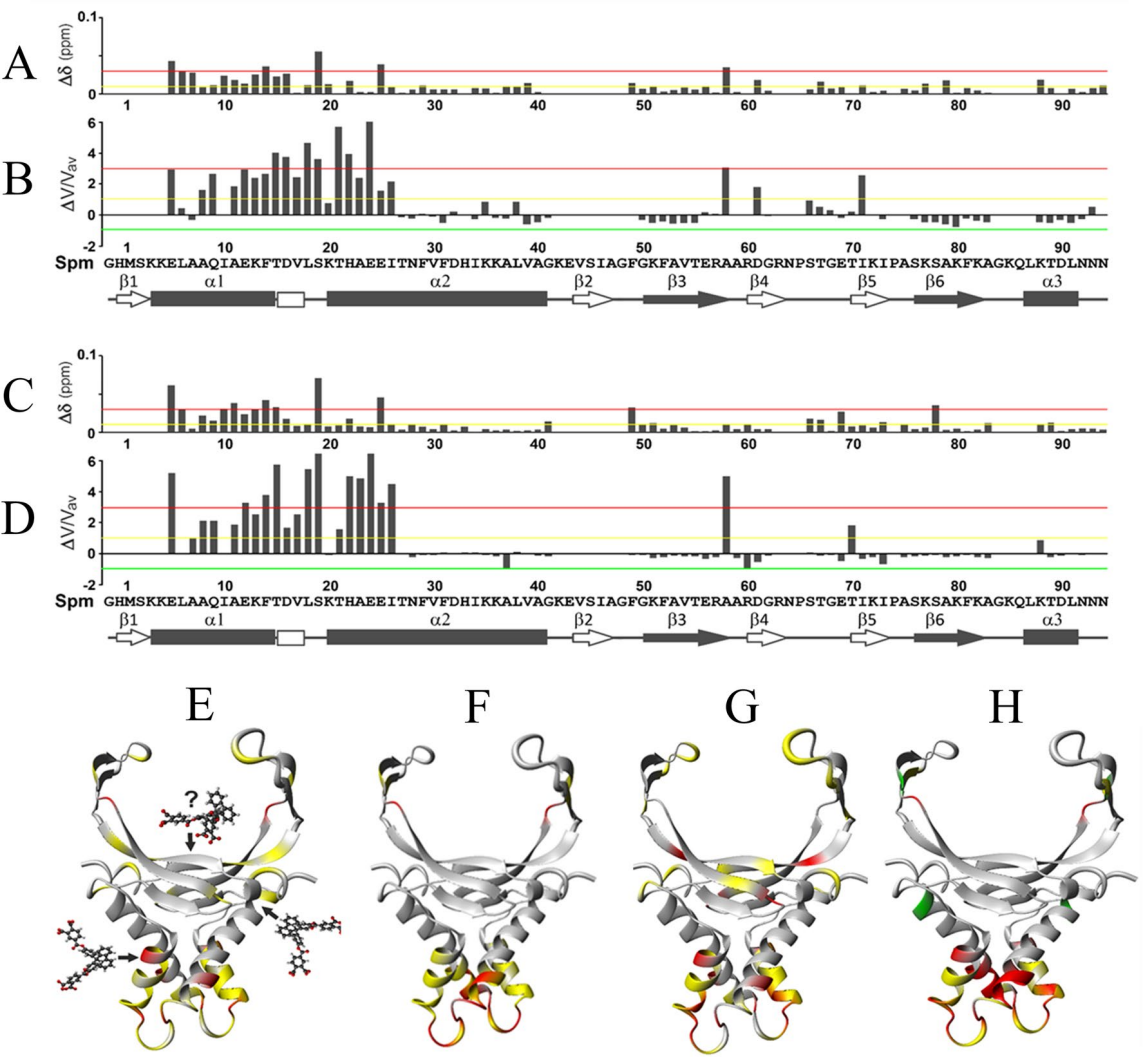
Monitoring of chemical shift perturbations (CSPs) and cross-peak intensity perturbations (CIPs) of backbone amide groups of the HUSpm dimer in its free form (**A**,**B**,**E**,**F**) and in complex with DS14 (**C**,**D**,**G**,**H**) upon BDF4 binding. Weighted CSPs (**A**,**C**,**E**,**G**) and normalized CIPs (**B**,**D**,**F**,**H**) of the HUSpm residues after binding BDF4 shown as diagrams (**A**–**D**) and their color-coded mapping on ribbon models of the HUSpm dimer (**E**–**H**). Green, yellow and red lines in (**A**–**D**) are cutoff levels for the color-coding used in (**E**–**H**).

In all complexes (HUSpm + DS14, HUSpm + BDF4 and (HUSpm + DS14) + BDF4), significant and moderate amide shift perturbations were observed for some amide cross peaks of HUSpm (see Supplementary Figure S5). For all HUSpm residues with assigned backbone amide signals, quantifications of weighted chemical shift perturbations (CSPs) and normalized cross-peak intensity perturbations (CIPs) of the backbone amide groups are visualized by the graphical view (Figs. 3A,B, 4A–D) and the color-coded mapping on the HUSpm dimer ribbon structure (Figs. 3C,D, and 4E–H).

Increased CSP values are usually associated with local structural perturbations and changes in the local environment of the amide group of interest, whereas positive and negative CIP values, which result from the relative signal narrowing and broadening, respectively, can indicate changes in intramolecular local motions over a wide range of time scales from fast pico- to nanosecond movements to slow conformational transitions in a micro-millisecond time scale.

According to Fig. 3A,C, in case of HUSpm-DNA complex formation, the residues showing the most significant chemical shift changes belong to the C-terminal part of the protein and reside in the DNA-binding β‐saddle-like domain including the α3-helix (see Fig. 1A). This effect coincides with a model of HUSpm interaction with DS14 DNA taken from 1,000 ns MD simulations of the corresponding complex obtained by comparative modelling (Fig. 3E). A similar effect has been reported in the NMR study of the interaction of HU protein from *Helicobacter pylori* with 25 bp hairpin DNA, which was very similar in length to our 14 bp DNA-duplex^39^.

In addition, as expected upon DNA-binding, significantly increased CIPs (> 1 and > 3, *yellow* and *red* levels, respectively) due to NMR signal narrowing were observed for amide groups of residues belonging and adjacent to the β4- and β5-strands surrounding DNA-intercalating tips (Fig. 3B,D). This observation illustrates the transition of flexible DNA-binding arms from a free state characterized by conformational exchange and high-amplitude movement^37^ to a DNA-binding mode characterized by rapid low-amplitude movement that facilitates the capture of DNA and intercalation of tips in the DNA minor groove. Thus, in this case, the narrowing of the signal is primarily caused by a decrease in conformational diversity with slow transitions in the micro-and millisecond range due to the formation of a DNA–protein complex.

Notably, moderate negative CIPs (< − 1, *green* level) due to NMR signal broadening were identified in the opposite, N-terminal helical part of the HUSpm dimer probably owing to the appearance of slow conformational transition (on the micro-millisecond time scale undetectable by the 1,000 ns MD simulation). Thus, both CSP and CIP distribution patterns indicate that an allosteric (in scissor-like manner) rearrangement of the protein structure and redistribution of its backbone motions occur in HUSpm dimer upon DNA binding.

The addition of BDF4 to HUSpm caused opposite effects compared to those observed upon DNA binding (Fig. 4). When the stoichiometry of inhibitor to HUSpm dimer (either in free form or in complex with DNA) was about 1:1, significant CSPs (between 0.01 and 0.03, *yellow* level) were observed mainly for residues from the N-terminal α-helical part of the protein located in the α1-helix and in the N-terminal part of the α2-helix, whereas CSPs were not significant for the majority of residues from the C-terminal DNA-binding domain (Fig. 4A,C,E,G). In both cases, the most significant CSPs (> 0.03, *red* level) were observed for several residues from the α1- and α2-helices, and for one and two residues of the DNA-binding domain in case of HUSpm + BDF4 and (HUSpm + DS14) + BDF4 complexes, respectively (Fig. 4A,C,E,G). These three residues are F49 from DS region of HUSpm (see Fig. 1A), A58 from the C-terminal end of the β3-strand and S78 from the β6-strand. The two former residues belong to the dimer interface and the latter to the stable core of DNA-binding domain. In this regard, we should note that residues with affected chemical shifts might be involved in the interaction with BDF4 either directly or indirectly by participating in the local or global conformational rearrangements in response to inhibitor binding.

Moreover, NMR signal intensity analysis revealed that more pronounced changes of CIPs (> 1 and > 3, *yellow* and *red* levels, respectively) were also observed for residues from the α1 and α2-helices upon BDF4 interaction with both free HUSpm and a HUSpm + DS14 complex (Fig. 4B,D,F,H). These significant increases of CIPs indicated that residues from the α1-helix and N-terminal part of the α2-helix underwent fast pico- to nanosecond time scale motions resulting in local enhancement of backbone flexibility in the HUSpm α-helical domain.

Thus, both CSPs and CIPs distribution patterns revealed that interaction with BDF4 caused an opposite effect on structural and motional features of HUSpm compared to that occurring upon DS14 binding, which indicates low probability of BDF4 interactions with the core of the DNA-binding domain.

**Figure 5:**
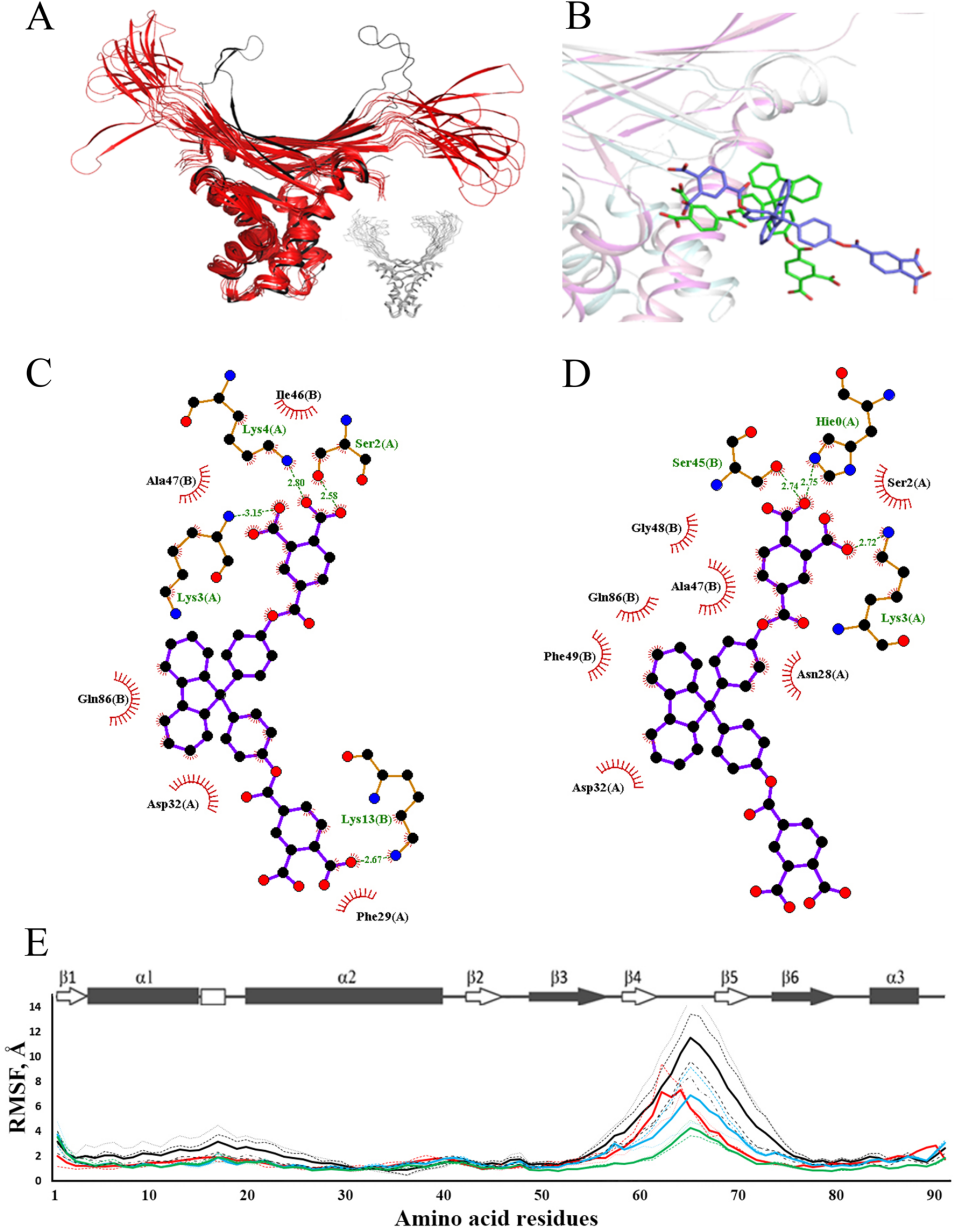
Side-site model of BDF4 binding to HUSpm and MD analysis of the corresponding complex. (**A**) Superposition of MD models (colored red) taken from the MD trajectory with an interval of 100 ns and an energy-minimized model of HUSpm (colored black) in complex with BDF4 bound to the side-site. Only the protein is shown to illustrate the increased movement of the DNA-binding domain. A similar picture obtained for free HUSpm (colored gray) has been inserted for comparison. (**B**) Position of BDF4 at the beginning (green colored inhibitor) and end (blue colored inhibitor) of MD simulation. The protein is shown as a cartoon model, one monomer is colored in magenta and another in gray. Only one BDF4 molecule is shown for clarity. (**C**,**D**) Polar and hydrophobic interactions of BDF4 molecule with HUSpm amino acid residues at the beginning (**C**) and end (**D**) of MD simulation. The diagrams were obtained with LigPlot software. (**E**) RMSF values of the Cα-atoms (in Å) derived from 1,000 ns MD trajectories obtained for a free HUSpm dimer (blue lines), HUSpm interacting with DS14 (green lines), and HUSpm interacting with BDF4, where the inhibitor targeted either the core of the DNA-binding domain (red lines) or overlaid the dimer interface between the α-helical domain of one monomer and the side portion of the DNA-binding domain of another monomer (black lines). Discontinuous and solid lines show the RMSF values obtained from independent MD experiments and the corresponding mean values, respectively. Secondary structure distribution derived from NMR data^37^ is shown above.

Since NMR-derived data contradicted with docking prediction about BDF4 interaction with the core of the DNA-binding domain of HUSpm, a new docking experiment was performed, in which the whole surface area of the HUSpm dimer was the target for BDF4 inhibitor; then the modelled complex was studied by Molecular Dynamics (Fig. 5). The available NMR data indicated the localization of the main conformational-dynamic changes in HUSpm, caused by the binding of the inhibitor, in the α-helical domain of the protein, but did not provide more detailed information about the specific localization site of the inhibitor at the recognition stage and in the final bound state. Therefore, docking and subsequent MD simulations were performed without using any NMR constraints.

As a result, new binding sites located on the opposite sides of the HUSpm dimer were found. Figure 5A illustrates the conformational dynamics of the HUSpm dimer with two BDF4 molecules (not shown in the Figure) bound to the new sites in the course of 1,000 ns MD simulation. Figure 5B–D shows the binding modes and amino acid residues interacting with the inhibitor at the beginning and end of the simulation. According to Fig. 5C,D, the new binding site consists of residues from α1- and α2-helices of one monomer and two loops adjacent to the core of the DNA-binding β-saddle (β2-loop-β3 and β6-loop-α3) of another monomer. Both the hydrophobic core and carboxyl groups of BDF4 were driving forces of the inhibitor binding, which coincides with the fact that other BDFs (Supplementary Table S2) are mainly inactive.

MD trajectories obtained for two models of HUSpm + BDF4 complex (which were dubbed top-site and side-site for the old and new model, respectively) were compared to those obtained for HUSpm and its complex with a DS14 DNA duplex. Figure 5E shows root mean square fluctuations (RMSF) values of Cα-atoms for all HUSpm residues derived from calculated MD trajectories. Increased RMSF values indicate enhanced flexibility of the protein backbone. The enhanced flexibility of residues located at the N-terminal part of the protein on the α1-helix and in the beginning of the α2-helix was clearly visible at a nano-microsecond MD timescale of the side-site model (black line), and was in good agreement with results of NMR signal intensity analysis (Fig. 4B,F). Moreover, MD simulations of the side-site model demonstrated that such a mode of BDF4 binding causes a significant increase of the flexibility of DNA-binding arms and moderately increases the flexibility of the stable part of DNA-binding domain connecting the flexible arms, as compared to the free HUSpm dimer (Fig. 5A,E). These effects are opposite to those associated with DNA-binding (reduced RMSF values for residues from the DNA-binding domain), which reflects a decreased amplitude of motion of the DNA-binding arms.

### Alanine substitution of the basic amino acid residues playing key roles in DNA binding did not affect HUSpm sensitivity to bisphenol derivatives of fluorene

To confirm or definitively disprove the probability of BDF4 binding to the HUSpm DNA-binding surface (top-site model), mutagenesis of amino acid residues presumably playing key roles in this binding mode was employed. The residues R57, K80 and K88 from the DNA-binding β-cleft were chosen for alanine substitution because they form polar contacts with BDF4 carboxyl groups according to the top-site model (Fig. 1D,E). According to^35^, alanine substitution of R55 (R57 in HUSpm) and K86 (K88 in HUSpm) led to reduced binding of both DNA and stilbene derivative inhibitors to HUMtb. Our previous analysis of conserved residues in the HU protein characteristic core^5^ showed that the residues R57 and K88, but not K80, were conserved among HU proteins (Supplementary Figure S4).

**Figure 6:**
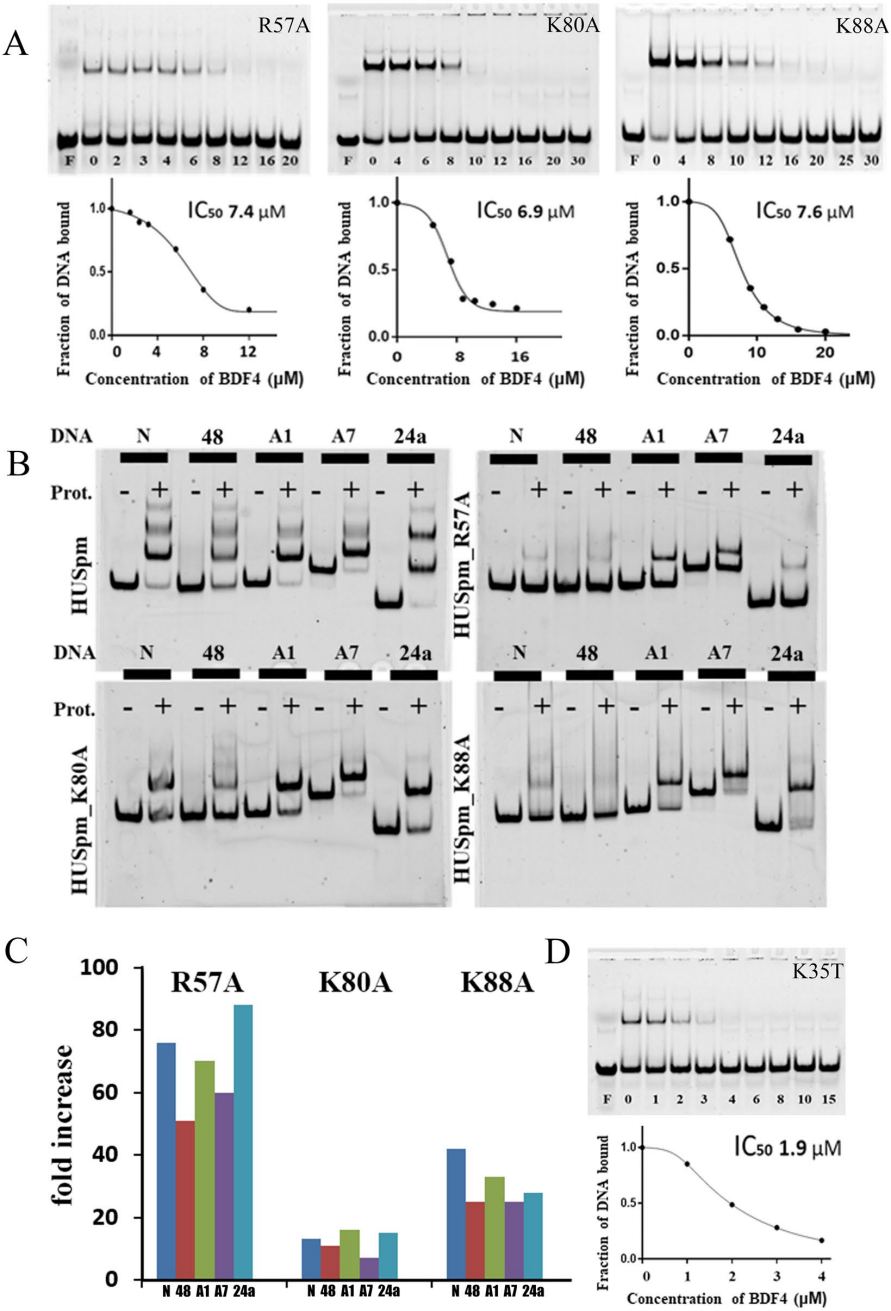
Disruptions in the DNA binding site did not influence the HUSpm sensitivity to BDF4. (**A**) Alanine substitutions of the positively charged amino acid residues located in the HUSpm β-saddle core does not affect HUSpm sensitivity to BDF4 in EMSA performed with the DS24a duplex; corresponding inhibition curves and IC_50_ values are shown. The mutant proteins were used in enhanced concentrations because of their low affinities to DNA: 100 nM for HUSpm_K80A and HUSpm_K88A, 200 mM for HUSpm_R57A. (**B**) Effects of the same mutations on the DNA binding properties of HUSpm. HUSpm (100 nM) and its mutant variants in concentrations indicated above were mixed with 5′-labelled DNA in a buffer containing 150 mM NaCl; the bound and free DNA were resolved in a polyacrylamide gel under nondenaturing conditions. DNA duplexes used are denoted at the top of the gel: N, nicked DNA; 48, DS48; A1, DS24a and A7, duplexes of different length, containing one or seven non-paired adenines in one strand. (**C**) Dissociation constants (k_d_) of the corresponding complexes normalized to those of HUSpm wild type. (**D**) The K35T substitution increases efficacy of BDF4. Inhibition of the HUSpm_K35T–DNA binding by BDF4 determined in EMSA, corresponding inhibition curve and IC_50_ value.

EMSA demonstrated that none of the three mutations in the β-saddle core significantly affected HUSpm sensitivity to BDF4 inhibitor (Fig. 6A, Table 1). In contrast, each of the mutant proteins bound various DNA structures with reduced affinities as compared to wild-type HUSpm (Fig. 6B,C and Table 2). Different DNA duplexes (Supplementary Table S4) including both canonical double stranded DNA (48) and distorted DNA structures of various length, carrying either nick (N) or an insertion of one and seven adenines in one DNA strand (A1, 24a and A7, respectively) were used in EMSA. To determine the affinities of each HUSpm mutant to different DNAs, the dissociation constants (k_d_) of observed complexes were calculated as described in^13,16^. Figure 6C shows that HUSpm_R57A has the weakest DNA binding (the increase of k_d_ values was between 51 and 88 folds for complexes with different DNAs over those of HUSpm), K88A and K80A mutations cause lesser effects (the increase of k_d_ values was between 25 and 42 folds and between 7 and 16 folds, respectively).

**Table 2:** Dissociation constants (K_d_ in nM, average ± SD, n = 2) for HUSpm wild type and HUSpm bearing mutations R57A, K80A and K88A binding to various DNA structures calculated from EMSA experiments (see Fig. 5B legend for a description of the DNA sequences).

DNA	HUSpm wild type	HUSpm_R57A	HUSpm_K80A	HUSpm_K88A
Nick (N)	5.2 ± 1.1	398 ± 45	69 ± 20	220 ± 40
DS48 (48)	18.6 ± 4.8	788 ± 100	163 ± 27	390 ± 55
A1 bulge (A1)	2.5 ± 0.6	175 ± 25	41 ± 8	83 ± 15
A7 bulge (A7)	2.0 ± 0.5	121 ± 15	13 ± 3	51 ± 8
DS24a (24a)	2.6 ± 0.6	230 ± 30	40 ± 10	84 ± 20

Since alanine substitutions of basic residues that reside in the HUSpm DNA-binding cleft and participate in DNA binding did not interfere with HUSpm interactions with BDF4, we analyzed effects of amino acid perturbations in the HUSpm dimer interface. Amino acid residues (F14, F29, F31 and K35) of HUSpm α-helical body contributing to the HUSpm dimer stabilization and maintaining its high thermal stability were identified earlier using structural analysis and site directed mutagenesis^9^. Phenylalanines are located in aromatic clusters strengthening the hydrophobic core of HUSpm dimer interface^36^ and K35 participates in intermolecular hydrogen bond formation with G48 that connects the α2-helix of one monomer with the loop between β2 and β3 strands of another monomer. This β2-loop-β3 pattern appears to be one of the most conserved regions of HU proteins and is named the dimerization signal (DS) (see Fig. 1A) for its role in the HU dimer formation and stabilization^8^. Thus, previously obtained HUSpm mutants (F14A, F29A, F31A and K35T) were employed for inhibitor evaluation. The result of EMSA performed with the DS24a duplex and HUSpm_K35T is shown in Fig. 6D, wherein the inhibition curve indicates that K35T substitution enhances sensitivity to BDF4; since about threefold decrease of the IC_50_ value was observed as compared to wild type HUSpm (Table 1). Other mutations did not cause any significant effects (data not shown). It is conceivable that disruption of the intermolecular H-bond connecting DS of one monomer to α2-helix of another monomer and the corresponding weakening of the dimer interface facilitates inhibitor action.

## Discussion

The general idea of this work is based on a previous study of Bhowmick and colleagues who reported successful development of a new chemical probe perturbing the DNA-binding function of HU protein from *M. tuberculosis* (HUMtb)^35^. The authors have performed very thorough analysis of the DNA-binding domain of HUMtb and suggested that the core or the base of the saddle-like DNA-binding domain (Fig. 1A) is the most “drugable” portion of HU proteins. Thus, following the methodology proposed in^35^ we conducted virtual screening of one million compounds from the Vitas-M laboratory chemical library collection against a high-resolution spatial structure of HUSpm and identified HU protein inhibitors of a chemical nature drastically different from previously described trans-stilbene derivatives^35^.

The main objects of this study, previously uncharacterized bisphenol derivatives of fluorene (BDF4, 5 and 6, see Supplementary Table S3), inhibit the DNA-binding properties of three tested HU proteins (Fig. 2A) and, as we strongly believe, should be active against any protein of this class. As we have shown, they also inhibit the growth of the simplest bacteria such as Mycoplasma spp. (Fig. 2B), where HU is the only NAP available; at the same time BDFs do not affect the growth of more complex bacteria, such as *E. coli*, which is consistent with the compensatory role of other NAPs in case of HU protein withdrawal. Notably, trans-stilbene derivatives inhibit growth of M. *tuberculosis*, whose genome has a remarkable under-representation of NAPs, but do not cause growth inhibition of *E. coli* and *M. smegmatis*^35^.

Further analysis was focused on the protein-inhibitor interaction and was carried out using the most potent and soluble inhibitor (BDF4). In addition to the high-resolution crystal structure of HUSpm (PDB ID 5L8Z^9^) that was used as a target for the virtual screening, HUSpm solution structure (PDB ID 5OGU^37^) obtained by means of NMR spectroscopy was used for a comparative analysis of the effects of BDF4 and DNA binding on the conformational dynamics of the target protein. A short canonical double-stranded DNA duplex (DS14) was used as model DNA in the NMR experiment. Analysis of the crystal structures (PDB ID 1P51, 1P71, 1P78, 2NP2 and 4QJU) obtained for HU proteins from cyanobacteria *Anabaena*, *Borrelia burgdorferi* and *Staphylococcus aureus*^7,40,41^ in complexes with DNA of various lengths indicated that such short DNA had to interact directly only with amino acid residues from the DNA-binding β-saddle domain including C-terminal α3-helix. The same mode of interaction was observed in a NMR study of HU protein from *H. pylori* in complex with 25 bp harpin DNA^39^. Thus, since BDF4 was designed and thought to recognize the core of the DNA-binding platform, chemical shift and cross-peak intensity perturbations in the NMR spectra of HUSpm titrated with BDF4 were supposed to be somehow similar to the CSPs and CIPs observed in case of the HUSpm + DS14 complex. In fact, addition of BDF4 to HUSpm caused the opposite effects (described in the Results) relatively to those expected for and observed in the NMR spectra of HUSpm after addition of DS14 (Figs. 3 and 4).

Since BDF4 specificity to the DNA binding site at the base of the β-saddle was not confirmed by NMR spectroscopy, we performed a new docking experiment, in which the whole surface area of HUSpm was the target for the inhibitor. Two symmetrical binding sites located on opposite side-surfaces of HUSpm were identified; each site overlapped a dimer interface between the α-helical domain of one monomer and the side portion of the DNA-binding domain of another monomer (Fig. 5B–D). Conformational dynamics of the HUSpm dimer observed during MD simulations of this model (Fig. 5E) coincided mostly with chemical shift and cross-peak intensity perturbations detected by NMR spectroscopy.

To distinguish finally between the initially proposed BDF4 binding site in the core of the β-saddle domain and the new site, we performed site-directed mutagenesis. It demonstrated that alanine substitutions of basic DNA-interacting residues^7, 39–41^ which reside in the core of DNA-binding domain and presumably participate in inhibitor binding (Fig. 1D,E), actually disturb DNA-binding, but do not affect the activity of BDF4 (Fig. 6A–C). This result confirmed the NMR-based conclusion about different locations of BDF4 and DS14 binding sites on the HUSpm dimer.

At the same time, K35T substitution, which weakened the HUSpm dimerization interface by disturbing the hydrogen bond between the β2-loop-β3 of one monomer with the α2-helix of another monomer^9^, led to an increase of HUSpm sensitivity to BDF4 (Fig. 6D). It is conceivable that BDF4 binding to the site overlapping the aforementioned area of the dimer interface (Fig. 5C,D) initiated destructive changes in the dimerization interface. Moreover, according to the MD study, BDF4 binding to the side-surface of HUSpm significantly influences flexibility of the DNA-binding domain (Fig. 5E).

Thus, the inhibitor binding in the site located at the interface between the α-helical domain of one monomer and the DNA-binding domain of another monomer causes a conformational rearrangement of the latter. We strongly believe that this conformational rearrangement fixes the HU dimer in a defective DNA binding mode, in which the electrostatic interactions between DNA and protein are not supported by proper conformational adaptation of the DNA-binding domain (1), the strength of the DNA binding is lowered to a level not detectable in EMSA (2), and therefore the protein is not able to perform its biological function as the architect of the bacterial genome (3).

We suggest that any inhibitors targeting the DNA-binding surface may mimic and, to be precise, partially mimic the electrostatic part of DNA-HU protein interaction. This gives micromolar dissociation constants of protein-inhibitor complexe^35,36^ vs*.* nanomolar dissociation constants for HU protein-DNA complexes^5,35,41^ (for k_d_ values of the HUSpm-DNA complexes see Table 2). Thus, high potency of inhibitors targeting the DNA-binding surface in vivo may be questionable. Meanwhile, as we report here, there is another way to disrupt DNA-binding features of HU proteins without direct competition for the binding site between the inhibitor and native HU protein “ligand”, such as DNA. Both MD simulation of the protein-inhibitor complexes and NMR study performed here suggest that the α-helical domain of HU protein is “druggable” by so called “interface inhibitors”, which are able to disturb the DNA binding allosterically, and bisphenol derivatives of fluorene are the first examples of such inhibitors. We believe that new molecules with similar features are going to be identified soon, since targeting the interface area of the HU proteins’ α-helical body may be less challenging than targeting the large and adaptive DNA-binding surface.

## Conclusion

Here we report bisphenol derivatives of fluorene (BDFs) as a new type of chemical probes targeting histone-like HU proteins, which are global regulators of bacterial nucleoids, through dimer interface perturbation. BDFs were found by virtual screening targeting the DNA-binding surface in the core of the β-saddle-like domain of HU protein from *S. melliferum.* They inhibited the DNA binding properties of three tested HU proteins from mycoplasmas *S. melliferum* and *M gallicepticum,* and from enterobacteria *E. coli* with IC_50_ values within the range from 5 to 10 µm. In addition, BDFs demonstrated antimicrobial activity against mycoplasmas, but not against *E. coli*, which was consistent with compensatory role of other NAPs in the higher bacteria. At the same time, NMR spectroscopy complemented with Molecular Dynamics and site-directed mutagenesis indicated that the dimer interface between the α-helical domain of one monomer and the β-saddle domain of another monomer is the actual site of inhibitor intervention. Thus, we propose that the allosteric inhibition mode reported here, which does not require direct competition with DNA for the binding site, should be considered during development of small molecule inhibitors of nucleoid-associated proteins as well as other type of DNA-binding multimeric proteins.

## Methods

### Compound library and virtual screening

Structures of compounds were downloaded from the chemical library collection of Vitas-M laboratory (Causeway Bay, Hong Kong) (https://vitasmlab.biz/). Virtual screening was performed using Lead Finder software package (MolTech LLC, Moscow, Russia^42^, the dock_filter protocol^43^ was applied to filter the subset of ligands interacting simultaneously with both protein “arms” and the beta-saddle so as to screen for compounds with the best abilities to bind to HUSpm’s DNA-binding cleft. Ligands were processed with OpenBabel 2.3.2 software^44^ to remove counterions, to generate physiological charge states and tautomers wherever applicable. Energy-minimized 3D structures for all compounds were generated and subjected to the molecular docking.

To prepare the target protein (PDB ID 5L8Z^9^) for docking, unresolved residues were restored using Modeller softwar^45^. Hydrogen atoms were added to the HUSpm spatial structure using Build Model unit of the Lead Finder software package. Visualization of protein-inhibitor complexes and intermolecular interaction was performed using the VMD^46^, LigPlot^47^ and the PyMOL Molecular Graphics System, Version 1.9.0.0 (Schrödinger, LLC, New York, NY, USA).

### Docking to the whole surface of HUSpm

Virtual screening and molecular docking were performed using the Lead Finder software package (MolTech LLC, Moscow, Russia) with built-in standard precision preset using the approach developed in the previous work^48^. Searching for potential binding sites was performed in the whole surface of HUSpm as follows. A set of 50 energy grids evenly distributed around the protein surface was calculated, and then the test ligand was docked into each grid five times. Binding free energies calculated in each run were averaged in each energy grid. A potential binding site was considered to be found if an average calculated binding free energy for any given grid was significantly lower (p < 0.05) than the overall average.

The final compounds obtained by screening were re-docked using the extra precision Lead Finder preset to select the molecules with the most desirable sets of interactions.

### Homology modelling and molecular dynamics calculations

Preparation of the HUSpm + DS14 protein-DNA complex was carried out using the COOT interactive graphics program^49^. The structure of HU protein from *B. burgdorferi* complexed with DNA (PDB ID 2np2) was superimposed with the HUSpm model, and the coordinates of the DNA were transferred to the HUSpm model. After that, excess nucleotides were removed from the model, and the remaining nucleotides were replaced by those from the DS14 oligonucleotide duplex (Supplementary Table S4).

MD simulations were carried out for free HUSpm and its complexes with DS14 DNA (Supplementary Table S4) and BDF4 (Supplementary Table S3) using the GROMACS 2019.2 simulation package^50^. OPLS-AA/L force field^51^ was used with a rectangular model box with a minimum distance from the solute molecule of 10 Å and the TIP3P water model. Sodium and chloride ions were added to the system for equalization of the total charge and adjustment of the ion strength to 0.15 M. A topology for BDF4 was created using the LigParGen service^52^. Charges were computed using the RESP procedure implemented in Antechamber^53^ based on HF/6-31G QM-optimized geometry and electron density. The simulation protocol included 5,000 steps of steepest descent energy minimization, 100 ps NVT equilibration, and 1,000 ps NPT equilibration. The pressure and temperature of the system were controlled, where appropriate, with the Parinello-Rahman barostat and the V-rescale thermostat, respectively. All calculations were performed using the supercomputer of NRC “Kurchatov Institute”. The simulations were repeated two or three times with similar results.

### Purification of the recombinant HU proteins

Cloning, expression and purification of the recombinant HU proteins have been described in our previous works for HUSpm^54^, HUMgal^55^, and HUEc^5^. Preparation of samples for heteronuclear NMR spectroscopy was carried out as described in^37,56,57^ with minor modifications. Briefly, ^15^N-labeled HUSpm was purified using Ni–NTA Superflow column (Qiagen, Hilden, Germany) and digested with TEV-protease (1 mg per 10 mg of protein). The 6xHisTev-tag and TEV-protease were removed by the second run of Ni–NTA affinity chromatography. After that, instead of size-exclusion chromatography, HUSpm was subjected to buffer exchange to 20 mM TrisHCl buffer pH 7.8 using PD10 desalting columns (GE Healthcare, Chicago, IL, USA) and loaded to the ResourceS cation exchange column (GE Healthcare, Chicago, IL, USA) equilibrated with the same buffer. The column was washed with the linear gradient of NaCl from 10 mM to 1 M. The major peak containing HUSpm was detected at NaCl concentration about 26%. This step prevented the proteolytic degradation previously observed during NMR spectroscopy. Finally, the protein solution was exchanged to 50 mM Na_2_HPO_4_/NaH_2_PO_4_ buffer containing 0.1 mM NaN_3_ in 9/1 H_2_O/D_2_O (v/v) mixture with pH 7.2, and subsequently it was concentrated to 20 mg/ml (which corresponded to 1 mM of HUSpm dimer) using a 3 kDa cutoff centrifugal filter device (Millipore, Burlington, MA, USA).

### Electrophoretic mobility shift assay (EMSA)

The fluorescent-labeled oligonucleotide duplexes described in Supplementary Table S4 were prepared by annealing labeled oligonucleotides, 5′-HEX-D48 or 5′-HEX-D24a, and corresponding non-labeled oligonucleotides. All oligonucleotides were synthesized by Evrogen (Moscow, Russia). The annealing was performed by incubating the oligonucleotides (10–50 μM) for 3 min at 90 °C in 20 mM Tris–HCl (pH 8.0) and 200 mM NaCl, and then allowing them to cool slowly (∼ 4 h) to 40 °C.

Binding of HU proteins to DNA was analyzed by electrophoretic mobility shift assays (EMSA) performed as described previously^5^. Different amounts of the HU proteins (30 nM HUSpm and HUSpm_K35T, 60 nM HUMgal and HUEc, 100 nM HUSpm_K80A and HUSpm_K88A, and 200 mM HUSpm_R57A) were incubated with DNA (10 nM) in 10 μl of the binding buffer (20 mM Tris–HCl pH 8.0, 7% glycerol, and 150 mM of NaCl) for 10 min. Then, the samples were loaded to pre-runned (125 V, 30 min) 10% non-denaturing PAAG, buffered with 100 mM Tris–borate, and electrophoretically separated (125 V, 90 min). For 48 bp DNA duplexes, 8% PAAG was used. Gels were visualized using BIO RAD Faros FX Molecular Imager (532 nm EX, 605 nm BP), and quantification was performed using Quantity One software. The dissociation constants (Kd) were calculated as described earlier^16^.

To evaluate the effects of inhibitors, 1 μl volumes of serial dilutions of tested compounds or DMSO were added to the preformed protein-DNA complexes. After five minute incubation, the assay was carried out as described above. Inhibition curves and IC_50_ values were obtained using GraphPad Prism 5 software. The figures showed representative results of an independent assay; the combined data from three independent experiments are shown in the tables.

### Heteronuclear NMR spectroscopy

The heteronuclear NMR experiments^58^ were recorded with uniformly ^15^N-labeled HUSpm (1 mM, dissolved in 50 mM Na_2_HPO_4_/NaH_2_PO_4_ buffer containing 0.1 mM NaN_3_ in 95/5 H_2_O/D_2_O (v/v) mixture with pH 7.2) at 30 °C on Varian NMR-system 700 MHz spectrometer (Varian-Agilent, USA) equipped with ^1^H/^13^C/^15^N gradient cryoprobe. The Resource Center of Molecular and Cell Biology of the Kurchatov complex of NBICS-technologies NRC “Kurchatov Institute” kindly provided access to the instrument. Two-dimensional heteronuclear ^1^H/^15^N–HSQC (heteronuclear single quantum correlation) and ^1^H/^15^N–TROSY (transverse relaxation-optimized spectroscopy, BEST version^59^) spectra were acquired to monitor the HUSpm interactions with DNA and BDF4. The spectra were analyzed using Computer Aided Resonance Assigment (CARA)^60^ based on the previously obtained HUSpm chemical shifts^37^.

For studying the interaction of HUSpm with DNA, 14 bp oligonucleotide duplexes DS14 (Supplementary Table S4) were prepared as described above (see EMSA section) at the 5 mM concentration in the aqueous 50 mM phosphate buffer of pH 7.2. The complex of 0.5 mM ^15^N‐labeled HUSpm with DS14 was prepared by mixing the protein with 20% molar excess of the DNA.

For studying the effects of inhibitor, BDF4 dissolved in DMSO (or DMSO only) was gradually added to ^15^N-labeled HUSpm (0.5 mM) either in apo-form or in complex with DS14 with the molar ratios of the protein dimers or protein-DNA complexes were 1/1.2 and 1/2.0, after which the high protein precipitation was observed. The addition of the similar amount of DMSO to the apo-form or complex with DS14 did not result in any changes in the NMR spectra.

For quantification of the changes in the NMR spectra due to the interactions of HUSpm with DS14 and BDF4, the weighted chemical shift perturbations (CSPs) and normalized cross-peak intensity perturbations (CIPs)of the backbone amide groups were estimated at protein-dimer/DNA-duplex and protein-dimer/inhibitor molar ratios of 1/1.2 (where the protein precipitation was not observed). The CSPs for backbone amide ^15^N^15^N^H^ and ^1^H^N^ resonances were calculated by the formula Δδ = [(Δ^1^H^N^)^2^ + (Δ^15^N^H^/5)^2^]^0.5^, where Δ^1^H^N^ and Δ^15^N^H^ are changes in the chemical shifts of backbone amide ^1^H and ^15^N, respectively, upon either DS14 or BDF4 binding to HUSpm, or BDF4 binding to HUSpm + DS14 complex. The CIPs for HUSpm backbone amides were calculated by the formula ΔI/I_av_ = I/I_av_ − I_ref_/I_ref_av_, where I and I_ref_ were the intensities of the amide cross-peaks in the pairs of the NMR spectra of (HUSpm + DS14 and HUSpm), (HUSpm + BDF4 and HUSpm) or (HUSpm + DS14 + BDF4 and HUSpm + DS14), which were normalized by the averaged “standard” cross-peak intensity I_av_ estimated along the region T27-K35 that undergoes minimal local flexibility of the protein backbone according to previous study^37^.

### Site-directed mutagenesis

Easy single-primer site-directed mutagenesis was performed as described in^61^. The synthetic oligonucleotide primers designed for the changing of amino acids (one primer for each mutant) and those designed for the selection of mutant clones are listed in the Supplementary Table S5. Eighteen cycles of PCR were performed with the template of the HUSpm-expressing plasmid using the Tersus Plus PCR kit (Evrogen, Moscow, Russia) according to the manufacturer’s recommendations. The PCR products were treated with DpnI endonuclease (Thermo Fisher Scientific, Massachusetts, United States), which digested the parental DNA template, and then transformed into *E. coli* Match1 competent cells. The mutant clones were selected by PCR performed directly on colonies using Taq DNA polymerase (Evrogen, Moscow, Russia) with check primers (Supplementary Table S5) and an appropriate T7 universal primer. Plasmid DNAs purified from mutant clones were sequenced to ensure the absence of random mutations associated with PCR. The expression and purification of mutant proteins were performed in the same manner as described for the wild type HUSpm. The purity of the mutants was estimated by SDS–PAGE with Coomassie staining, and the protein concentrations were measured using the Bicinchoninic Acid Protein Assay Kit (Sigma-Aldrich, St. Louis, USA).

### Inhibition of mycoplasma growth

The *S. melliferum* KC3 strain was kindly provided by Prof. G. Wroblewsky of the University of Rennes, France. The *M. gallisepticum* S6 strain was provided by Prof. S. N. Borkhsenius, Institute of Cytology, St. Petersburg, Russian Academy of Science. The bacteria were cultivated as described in^62^ without any specific aeration in covered flasks, for less than five passage. *M. gallisepticum S6* was cultivated in a modified Edward’s liquid medium (tryptose (20 g/l), Tris (3 g/l), NaCl (5 g/l), KCl (1.3 g/l), yeast dialysate (5%), heat inactivated (56 °C–30 min) horse serum (6%), glucose (0.5%) and penicillin 300U/ml, pH 7.5) at 37 °C for 20 h after 1:100 dilution. *S. melliferum* was cultivated in modified SP4 medum (tryptone (10 g/l), peptone (10 g/l), BHI (2.5 g/l), NaCl (4.5 g/l), yeast extract (7 g/l), sorbitol (70 g/l), horse serum (10%), sucrose (1%), fructose (0.1%), glucose (0.1%) and penicillin 300U/ml, pH 7.6) at 30 °C for 20 h after 1:10 dilution. All reagents were from Sigma-Aldrich (St. Louis, MO, USA).

For the broth micro-dilution assay, decreasing concentrations of BDFs dissolved in DMSO (or DMSO alone as a control) were mixed with the predetermined number of mycoplasma cells (10^4^/ml) in the 0.4 ml of standard broth medium containing pH indicator phenol red (red color at pH 7.5) in 48-well microplates. The plates were incubated until red color was changed to yellow (due to the pH decrease associated with lactate accumulation during mycoplasma growth) in the control (DMSO containing) wells. The MICs were considered as the lowest concentrations of BDFs that inhibited the color change at the time when it observed in the control wells. The experiments were repeated three times.

## Supplementary information

Supplementary Information 1.
