# Supplementary material for: Structure-based inhibitors targeting the alpha-helical domain of the Spiroplasma melliferum histone-like HU protein
Source: Sci Rep. 2020 Sep 15;10:15128. doi: 10.1038/s41598-020-72113-4 (PMC7493962; doi:10.1038/s41598-020-72113-4)
Supplement: Supplementary file 1 — Supplementary Information 1. [file 41598_2020_72113_MOESM1_ESM.docx]

**SUPPLEMENTARY MATERIALS**

**Structure-based inhibitors targeting the alpha-helical domain of the *Spiroplasma melliferum*** **histone-like HU protein**

**Yuliya K. Agapova^1#^, Dmitry A. Altukhov^1#^, Vladimir I. Timofeev^1,2^, Victor S. Stroylov^3^, Vitaly S. Mityanov ^3,4^, Dmitry** **A. Korzhenevskiy^1^, Anna V. Vlaskina^1^, Eugenia V. Smirnova^5^, Eduard V. Bocharov^5,6^, and Tatiana V. Rakitina^5^**

**List of supplementary materials**

**Table S1**  **2-3**

**Table S2**  **4-5**

**Table S3**  **6**

**Table S4**  **7**

**Table S5**  **8**

**Figure S1**  **9**

**Figure S2**  **10**

**Figure S3** **11**

**Figure S4** **12**

**Figure S5** **13**

Supplementary Methods. 14-15

Supplementary References. 16

**Supplementary Table S1.** List of compounds identified in the chemical collection of the Vitas-M laboratory (<https://vitasmlab.biz/>) by virtual screening.

| # | Molecular Structure | Molecular formula  Custom ID | ΔG  calc    kcal/  mol | IC_50_^1^ |
| --- | --- | --- | --- | --- |
| 1 |  | C_25_H_15_F_3_N_2_O_10_  STK367007 | -12.1 | No inhibition  up to 100 μM |
| 2 |  | С_14_H_14_O_6_S  STK733528 | -10.5 | No inhibition  up to 100 μM |
| 3 |  | C_23_H_26_N_2_O_9_S_4_  STL059355 | -12.3 | No inhibition  up to 100 μM |
| 4 |  | C_30_H_20_N_2_O_11_  STK156645 | -9.9 | No inhibition  up to 100 μM |
| 5 |  | C_59_H_36_N_2_O_10_  STK296333 | -8.4 | 6.5±0.9 μM  (6.2 ±0.5 μM) |

^1^ half-maximal inhibitory concentration of of HUSpm-DNA complex formation determined by EMSA performed with DS48 and DS24a (in parentless) DNA-duplexes (average±SD, n=3). DS24a was routinely used in further experiments.

Supplementary Table S2. List of bisphenol derivatives of fluorene identified in the screening collection of Chemical Block Ltd (www.chemical-block.com).

| **Custom ID**  **(compound #)^1^** | **Molecular structure** | **Molecular formula (Molecular mass)** | IC_50_**^2^** |
| --- | --- | --- | --- |
| А0345/0015874 |  | С_37_H_24_N_2_O_1_0S_2_  (720) | No inhibition  up to 100 μM |
| A0449/0020728 |  | С_73_H_44_N_2_O_10_  (1108) | > 100 μM |
| A0771/0036057 |  | С_39_H_24_F_2_O_4_  (594) | No inhibition  up to 100 μM |
| A0940/0043985  (BDF2) |  | C_55_H_30_N_4_O_12_  (938) | 29±5 μM |
| А0971/0045362  (BDF3) |  | C_59_H_36_N_2_O_10_  (932) | 36±6 μM |
| А2010/0084403 |  | C_43_H_28_N_10_O_4_  (748) | No inhibition  up to 100 μM |

^1^ as referred in the text

^2^ half-maximal inhibitory concentration of HUSpm-DNA (DS24a) complex formation determined by EMSA (average±SD, n=3)

Supplementary Table S3. List of synthesized bisphenol derivatives of fluorene (see Supplementary Figure 3 and Supplementary methods).

| **Compound number^1^** | **Molecular structure** | **Molecular formula**  **(Molecular mass)** | IC_50_^2^ |
| --- | --- | --- | --- |
| BDF4 |  | C_43_H_26_O_12_  (734) | 5.6±0.8 μM |
| BDF5 |  | C_57_H_40_N_2_O_10_  (912) | 6.3±0.7 μM |
| BDF6 |  | C_41_H_26_O_8_  (646) | 8.2±1.1 μM |

^1^ as referred in the text

^2^ half-maximal inhibitory concentration of HUSpm-DNA (DS24a) complex formation determined by EMSA (average±SD, n=3)

**Supplementary Table S4.** List of synthetic oligonucleotides designed for DNA-duplex formation.

| Duplex name  (short name) | Oligo  name | Sequence (from 5’ to 3’) | |
| --- | --- | --- | --- |
| DS48  (48) | D48_5 HEX | AGTCTAGAGTGCAGTTGAGTCCTTGCTACGACGGATCCCTTAGGTCAG |  |
|  | J48 | CTGACCTAAGGGATCCGTCGTAGCAAGGACTCAACTGCACTCTAGACT |  |
| DS24a  (24) | D24a_5  HEX | AGTCTAGAGTGC**A**AGTTGAGTCCTT |  |
|  | J24 | AAGGACTCAACTGCACTCTAGACT |  |
| Nick  (N) | D48_5 HEX + J24 + J23 | |  |
|  | J23 | CTGACCTAAGGGATCCGTCGTAG |  |
| A1 bulge  (A1) | J48+A1 | CTGACCTAAGGGATCCGTCGTAGCAAG**A**GACTCAACTGCACTCTAGACT |  |
| A7 bulge  (A7) | J48+A7 | CTGACCTAAGGGATCCGTCGTAGCAAG**AAAAAAA**GACTCAACTGCACTCTAGACT |  |
| DS14 | D14 | AGTCTAGAGTGCAG |  |
|  | J14 | CTGCACTCTAGACT |  |

**Supplementary Table S5.** List of synthetic oligonucleotide primers designed for amino acid changes and check primers for selection of mutant clones.

| Name | Sequence (from 5’ to 3’) | Tm, °C* | |
| --- | --- | --- | --- |
| R57A.F | AACCGAAAGAGCAGCA**GCC**GATGGAAAAGCAGGT  Check57A.R: ACCTGCTTTTCCATC**GGC** | 46 |  |
| K80A.R | ATGATC**G**AAAACA**GC**ATTTGTAATTTCTTCTGC  Check80A.F: GCAGAAGAAATTACAAAT**GC** | 44 |  |
| K88A.R | AAAGCAGGTAAACAATTA**GCC**ACTGATTTAAACAATAA  Check88A.R: ATTATTGTTTAAATCAGT**GGC** | 45 |  |

Mutations are shown in bold. Sequences following the mutations (3’ arms of primers) used for calculation of melting temperatures (Tm) are underlined. *Tm – was calculated by salt adjusted methods (<http://biotools.nubic.northwestern.edu/>OligoCalc.html) and used as the annealing temperature during PCR-based mutagenesis.

**Supplementary Figure S1.** Effects of compounds from the chemical collection of Vitas-M laboratory (Supplementary Table S1) on HUSpm-DNA complex formation determined in EMSA. Indicated amounts of the compounds were added to preformed HUSpm-DNA complexes (10 nM DS48 duplex + 30 nM HUSpm). Reaction products were resolved by 8% non-denaturing PAGE and visualized using BIO RAD Faros FX Molecular Imager (532 nm EX, 605 nm BP).


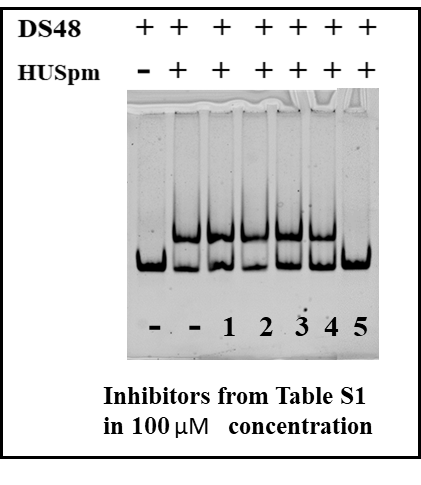


**Supplementary Figure S2.** Inhibition of HUSpm-DNA binding by BDF2 (**A**) and BDF3 (**B**) determined in EMSA, corresponding inhibition curves and IC_50_ values. Indicated amounts of the compounds were added to the preformed HUSpm-DNA complexes (10 nM DS24a duplex + 30 nM HUSpm). The reaction products were resolved by 10% non-denaturing PAGE and visualized using BIO RAD Faros FX Molecular Imager (532 nm EX, 605 nm BP).


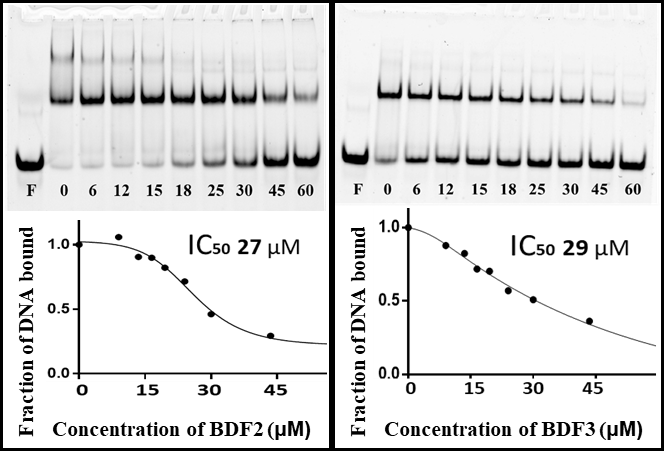


**A B**

**Supplementary Figure S3.** The synthesis scheme of the compounds BFD4, BFD5 and BFD56 (Supplementary Table 3). The synthesis details are described in Supplementary methods.

**Supplementary Figure S4.** Multiple sequence alignment of HUSpm and HU homologues with known 3D structures. The alignment was generated with ESPript, <http://espript.ibcp.fr> [63]. Highly conserved residues are highlighted in red, semiconserved residues are in red font. Secondary structure distributions derived from NMR data [37] are shown above the alignment. Non-colored secondary structure elements indicate non-stable (α-helix and β4/5-strands) as well as β1- and β2-strands, which were not clearly identified by NMR.


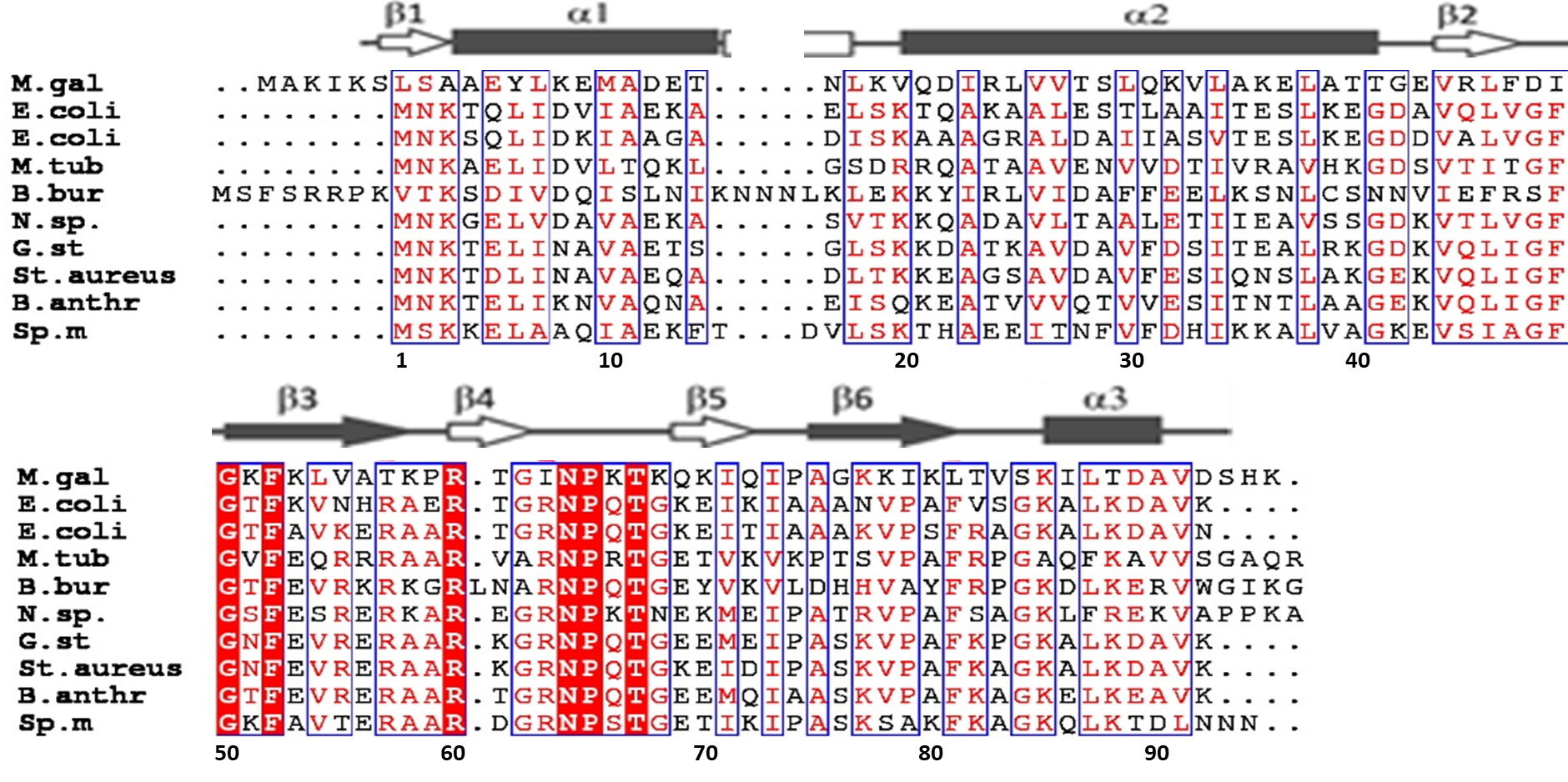


Supplementary Figure S5. HUSpm interactions with DS14 and BDF4 cause differential chemical shift and intensity perturbations of the amide cross-peaks as detected by means of high-resolution heteronuclear NMR spectroscopy. A-C Overlaid heteronuclear ^1^H/^15^N-TROSY spectra of the ^1^H^15^N-HUSpm in apo-form (blue) and in complexes (red) with DS14 at the protein-dimer/DNA-duplex molar ratio of 1.2 (A), with BDF4 at the protein-dimer/inhibitor molar ratio of 1.2 (B) and both, when BDF4 was added to preexisting HUSpm+DS14 complex at the complex/inhibitor molar ratio of 1.2 (C). D. ^1^H/^15^N-HSQC spectrum of the HUSpm dimer (рН 7.2, 30˚С) with resonance assignments of the backbone amide groups taken from [37]. Asterisks denote the assigned cross-peaks unambiguously attributed to minor HUSpm conformation. The ambiguous assignments are shown in *italic*.


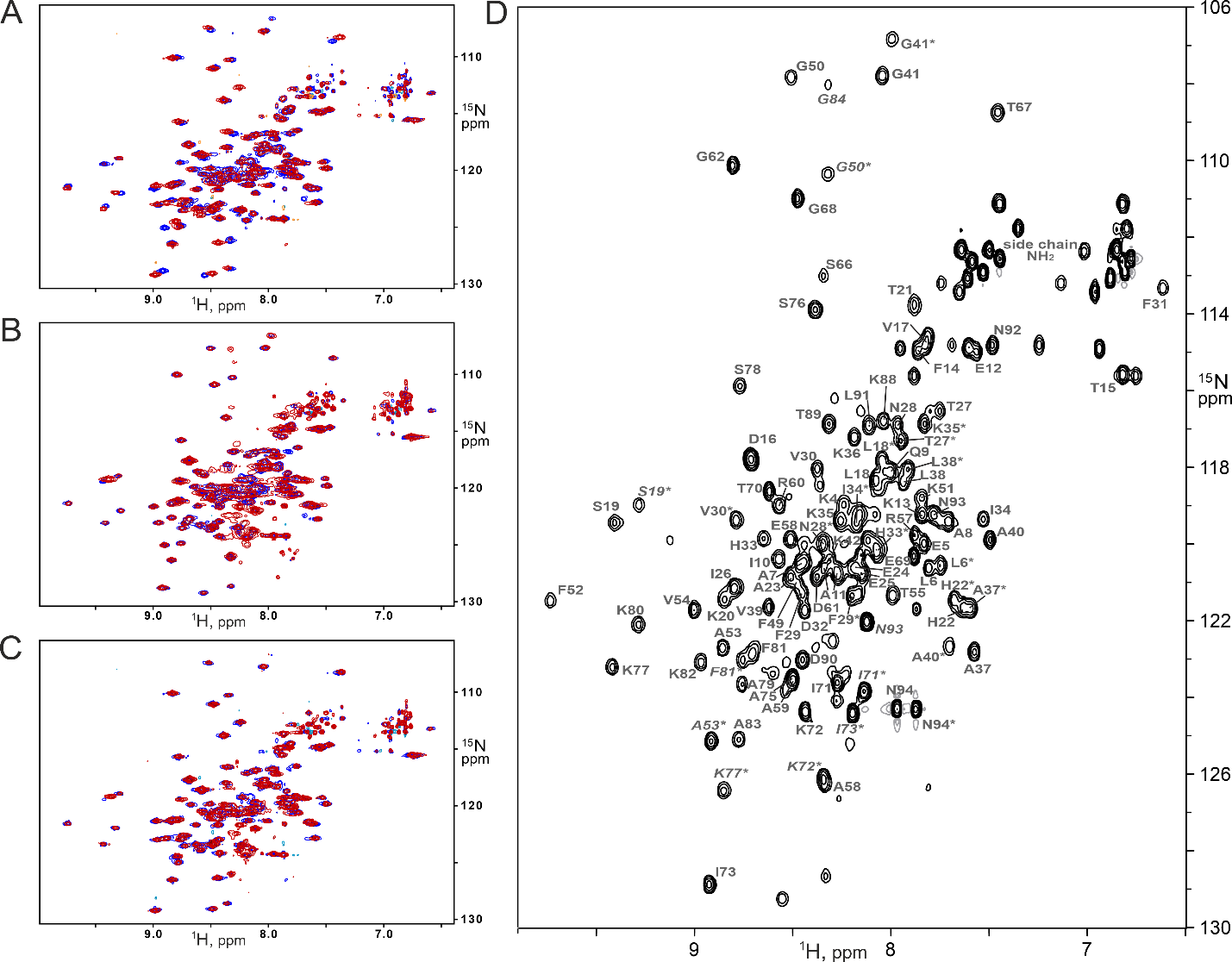


Supplementary Methods.

**Synthesis of BFD4, BFD5 and BFD6.**

**9H-fluoren-9-one (1)**. Dissolve 10.0 g (0.06 moles) of 9H-fluorene in 100 ml of glacial acetic acid. Heat to 80°С and gradually add aliquots of sodium bichromate dihydrate to a total of 18.0 g (0.06 moles) over 1.5 hours, keeping the temperature in the range 85-95°С. After addition of bichromate, incubate the reaction mixture at the same temperature for 2 more hours, then heat to the boiling point and keep boiling for 30 minutes. Cool the mixture and remove 60 to 70 milliliters of liquid by vacuum distillation. Add the residue to 400 ml of distilled water, filter the resulting precipitate, wash it thoroughly with water, and dry under vacuum over NaOH. Purify the product by flash-chromatography on 60-mesh silica using benzene as an eluent. The final yield of 9H-fluoren-9-one is about 80% (8.7 g). The substance forms yellow crystals with m.p. between 60 and 62°С.

**4,4'-(9*H*-fluorene-9,9-diyl)diphenol (2).** Dissolve 5.0 g (0.028 moles) of 9H-fluoren-9-one and 7.8 g (0.084 moles) of phenol in 50 ml of CCl_4_ then add 10.8 ml boron trifluoride etherate and incubate the mixture for 72 hrs at room temperature. After that wash the reaction mixture with 100 ml distilled water, separate the organic phase, desiccate it over MgSO_4_ and concentrate by vacuum distillation. Subject the concentrate to flash chromatography (60 mesh silica, eluent: hexane-ethyl acetate mixture 2:1 (v/v)). Concentrate the eluate under vacuum and resuspend in 30 ml of benzene-hexane mixture (1:1 v/v), then a white pellet should appear. Wash the pellet sequentially with 20 ml of benzene-hexane mixture (2:1 v/v) and 20 ml of hexane then vacuum-dry over paraffin wax. 4,4'-(9*H*-fluorene-9,9-diyl)diphenol forms white powder with m.p. 232-233 °С, and the final yield is 81%. Note that earlier works report m.p. 224°С [64].

**4,4'-(9*H*-fluorene-9,9-diyl)-bis(4,1-phenylene)-bis(1,3-dioxo-1,3-dihydro-isobenzofuran-5-carboxylate) (3)**. Place a solution of the Compound 4 in anhydrous THF (6.0 g (0.028 moles) in 15 ml) into a reaction flask, blow it with argon and add dropwise the solution containing 5.0 g (0.014 moles) of the Compound 2 and 3.3 g (0.042 moles) of pyridine in 25 ml of anhydrous THF under intense stirring. Keep the reaction temperature at 0°С and incubate the mixture for 3 more hours, then for 24 hours at RT. After incubation filter the mixture, wash the precipitate with small amount of anhydrous THF and evaporate the filtrate to dryness under vacuum, obtaining a cream-colored powder of the Compound 3 with 99% yield.

Element analysis calculate for (C_43_H_22_O_10_) C 73.92, H 3.17; Found C 73.68, H 3.36.

IR (KBr) at cm^-1^: 3 065 (C-H aromatic), 1863 and 1782 (C=O), 1742 (C=O), 1502.

^1^H NMR (CDCl_3_, δ in ppm ): 8.78 (s, 2H), 8.68(d, 2H), 8.15 (d, 2H), 7.80 (d, 2H), 7.41 (m, 4H),7.33 (m, 6H), 7.14 (d, 4H).

**1,3-dioxo-1,3-dihydro-isobenzofuran-5-carbonyl chloride (4)**. Dissolve 5.0 g (0.026 moles) of trimellitic anhydride in 3.1 ml (0.043 moles) of thionyl chloride, add several drops of dimethyl formamide and boil for 2.5 hrs under intense stirring. Cool and remove thionyl chloride by vacuum distillation, treat the rest with hexane to obtain a fawn-colored powder of the Compound 4 with 70% yield. Measured m.p. value of the product appears to be 65 vs. 66 °С, as previously reported [65].

**4,4'-[4,4'-(9*H*-fluorene-9,9-diyl)-bis(4,1-phenylene)]-bis(oxy)-bis(oxomethylene)diphthalic acid (5) (BDF4)**. Mix 200 mg (0.29 mmoles) of the Compound 3 with 5 ml of water and 0.5 ml pyridine, stir for 0.5 h, then adjust pH to 5 with 1M HCl solution, filter the precipitate and wash it with water. The yield of the Compound 5 is 60% (120 mg).

**4,5'-[4,4'-(9*H*-fluorene-9,9-diyl)bis(4,1-phenylene)]-bis(oxy)-bis(oxomethylene)-bis[2-(m-tolylcarbamoyl)-benzoic acid], mixture of regioisomers (6) (BDF5).** Dissolve 200 mg (0.29 mmoles) of the Compound 3 in 1 ml of DMSO and add 31 mg (0.30 mmoles) of *m-*toluidine. Stir the reaction mixture for 1 h then spill into 5 ml of water. Filter the pellet formed and wash it with water. The pellet is Compound 6 and its yield should be 59% (135 mg).

**3,3’-[4,4'-(9*H*-fluorene-9,9-diyl)-bis(4,1-phenylene)]-bis(oxy)-bis(oxomethylene)-dibenzoic acid (8) (BDF6)**. Place the solution of 3.0 g (0.018 moles) of isophthaloyl dichloride in 25 ml of anhydrous THF to an ice-cooled reaction flask, blow it with argon and add dropwise the solution containing 3.2 g (0.009 moles) of the Compound 2 and 2.1 g (0.026 moles) of pyridine per 30 ml of THF under intense stirring. Keep the mass temperature between 5 and 10 °С during addition, then incubate it for 2 hrs at RT. Concentrate the mass by vacuum evaporation, mix the rest with 50 ml of water and incubate for 1h at 85-90°С. Filter the suspension formed, wash the pellet with water then boil in 50 ml of methanol. Filter again and wash with methanol. The resulting white powder is Compound 8 and the yield should be 50% (2.9 g). ^1^H NMR (DMSO-*d*_6_, δ in ppm): 8.60 (s, 2H), 8.36-8.25 (m, 4H), 7.98 (d, 2H, *J*=7.5Hz,), 7.83-7.71 (m, 2H), 7.54 (d, 2H, *J*=7.2Hz), 7.47-7.35 (m, 4H), 7.25 (s, 8H).

**Isophthaloyl dichlolride (7)**. Mix 5.0 g (0.030 moles) of isophthalic acid with 5.0 g (0.043 moles) of thionyl chloride, add several drops of dimethyl formamide and boil for 2.5 hrs under intense stirring. Cool the mass and remove thionyl chloride by vacuum distillation. Consider the rest as isophthaloyl dichloride with 95% purity and use it in further synthesis.

Supplementary References.

1. Robert, X. & Gouet, P. Deciphering key features in protein structures with the new ENDscript server. *Nucleic Acids Res.* **42**, W320–W324 (2014).
2. Morgan P.W. Aromatic Polyesterswith Lange Cross-Plsnsr Sustituentis. *Macromolecules*, **3**, 536-544(1970).
3. Ulrich H., Richter R. 4-Isocyanatophthalic anhydride. Novel difunctional monomer. *J. Org. Chem.*, **38**, 2557–2558 (1973).
